# STAT4-associated natural killer cell tolerance following liver transplantation

**DOI:** 10.1136/gutjnl-2015-309395

**Published:** 2016-02-17

**Authors:** K M Jamil, T J Hydes, K S Cheent, S A Cassidy, J A Traherne, J Jayaraman, J Trowsdale, G J Alexander, A-M Little, H McFarlane, M A Heneghan, M A Purbhoo, S I Khakoo

**Affiliations:** 1 Department of Hepatology, Imperial College, London, UK; 2 Department of Hepatology, Southampton University, Southampton, UK; 3 Department of Pathology, University of Cambridge, Cambridge, UK; 4 Department of Hepatology, Addenbrookes Hospital, Cambridge, UK; 5 Histocompatibility and Immunogenetics Service, Gartnavel General Hospital, Glasgow, UK; 6 Institute of Liver Studies, Kings College Hospital London, London, UK

**Keywords:** LIVER TRANSPLANTATION, IMMUNOLOGY IN HEPATOLOGY, TOLERANCE, HEPATITIS C, RNA EXPRESSION

## Abstract

**Objective:**

Natural killer (NK) cells are important mediators of liver inflammation in chronic liver disease. The aim of this study was to investigate why liver transplants (LTs) are not rejected by NK cells in the absence of human leukocyte antigen (HLA) matching, and to identify a tolerogenic NK cell phenotype.

**Design:**

Phenotypic and functional analyses on NK cells from 54 LT recipients were performed, and comparisons made with healthy controls. Further investigation was performed using gene expression analysis and donor:recipient HLA typing.

**Results:**

NK cells from non-HCV LT recipients were hypofunctional, with reduced expression of NKp46 (p<0.05) and NKp30 (p<0.001), reduced cytotoxicity (p<0.001) and interferon (IFN)-γ secretion (p<0.025). There was no segregation of this effect with HLA-C, and these functional changes were not observed in individuals with HCV. Microarray and RT-qPCR analysis demonstrated downregulation of STAT4 in NK cells from LT recipients (p<0.0001). Changes in the expression levels of the transcription factors *Helios* (p=0.06) and *Hobit* (p=0.07), which control NKp46 and IFNγ expression, respectively, were also detected. Hypofunctionality of NK cells was associated with impaired STAT4 phosphorylation and downregulation of the STAT4 target microRNA-155. Conversely in HCV-LT NK cell tolerance was reversed, consistent with the more aggressive outcome of LT for HCV.

**Conclusions:**

LT is associated with transcriptional and functional changes in NK cells, resulting in reduced activation. NK cell tolerance occurs upstream of major histocompatibility complex (MHC) class I mediated education, and is associated with deficient STAT4 phosphorylation. STAT4 therefore represents a potential therapeutic target to induce NK cell tolerance in liver disease.

**Significance of this study**

**What is already known about this subject?**

- Natural killer (NK) cell alloreactivity is well described in bone marrow transplantation.
- As HLA matching is not employed in liver transplantation (LT), NK cell mediated alloreactivity is predicted after transplantation. However LT does not require high levels of immunosuppression.
- Previous studies have provided conflicting data on the effect of HLA matching for NK cell ligands in LT.

**What are the new findings**

- After LT, recipient NK cells exhibit a tolerant phenotype, with downregulation of activating receptors, reduced cytotoxicity and cytokine production.
- NK cell tolerance is associated with perturbation of the IL-12/STAT4 signalling pathway
- There is no effect of HLA matching on NK cell activation.
- NK cell tolerance is partially reversed in HCV infection.

**How might it impact on clinical practice in the foreseeable future?**

- Our data identifies STAT4 as a therapeutic target for inducing NK cell tolerance which may be important for LT and autoimmune liver disease.

## Introduction

Liver transplantation (LT) is a successful procedure despite being performed in the context of MHC mismatching, and in comparison to other organ transplants the doses of immunosuppression required are relatively modest.^1–3^ Multiple studies have demonstrated the relative tolerogenic microenvironment of the liver to classical T cell mediated immunity.^4^ However the liver is rich in cells of the innate immune system and how tolerance operates at the level of innate immunity is unknown.

Natural killer (NK) cells are cytotoxic innate lymphocytes forming up to 30% of the intrahepatic lymphocyte population. They are important mediators of liver damage in viral and inflammatory liver disease.^5–9^ NK effector functions are controlled by a balance of activating and inhibitory signals. Of particular relevance to LT are the killer cell immunoglobulin-like receptors (KIRs), which have HLA class I ligands defined by a KIR binding motif at residues 77–80 of the HLA molecule. The KIR can be either activating or inhibitory, but in the context of transplantation it is the inhibitory receptor interactions with HLA that dominate. In this context it can be considered that NK cells are educated on recipient HLA class I, and if there is a mismatch and the donor does not express the same HLA ligands for inhibitory KIR as the recipient then alloreactivity should result. This is observed in haematopoietic stem cell transplantation, in which clinically important NK cell alloreactivity can affect outcome.^10^ Conversely, as LT is performed without HLA matching there is a strong potential for NK cell alloreactivity against the graft. However this is not observed clinically.

Previous genetic studies have reported either neutral or adverse effects of mismatching ligands for NK cells.^11–14^ Furthermore functional studies have not shed light on the mechanisms of NK cell tolerance to the allograft. A limited analysis detected changes in the frequencies of specific NK cell subpopulations in the 1st week following transplantation.^15^ For instance it has been shown that there is a reduction in the frequency of NKp30-positive NK cells in children but not in adults transplanted for HCV.^16^ ^17^ Furthermore the effects of calcineurin inhibitors on NK cell activity are not clear, with studies showing either neutral or immunosuppressive effects in vitro.^18–22^ Human NK cells originate in the bone marrow, but can have some degree of maturation in the peripheral lymphoid tissue.^23^ As the liver is rich in immature CD56^bright^ NK cells this raises the possibility that NK cells may also mature in the liver.^24^ Thus there are multiple levels at which NK cells may be affected by LT.

The concept of NK cell tolerance, in general, and specifically in the context of liver disease, is poorly understood. However as NK cells interact directly with T cells and dendritic cells, they may be important as a target to downregulate liver inflammation in autoimmune liver disease, such as primary biliary cirrhosis in which there is increased NK cell cytotoxicity.^7^ Therefore in order to understand a potential mechanism for NK cell tolerance we have undertaken a cross-sectional study of individuals following LT.

## Materials and methods

### Patient recruitment

The study was conducted according to the Declaration of Helsinki. Patients were recruited from the outpatient clinics at Kings College Hospital, London and St Mary's Hospital, London. Demographic and clinical data are detailed in table 1.

**Table 1:** Demographic and clinical data

	Transplants		Healthy controls		p Value
Total, n	54		31		
Male, n (%)	32	(59.3)	18	(58.1)	NS
Age, years (SD)	60	(9.47)	41	(13.4)	<0.0001
Aetiology, n (%)					
HCV	18	(33.3)			
ALD	7	(13.0)			
PSC	7	(13.0)			
PBC	6	(11.1)			
ALF	5	(9.3)			
Cryptogenic/NASH	3	(5.6)			
Subacute	2	(3.7)			
AIH	1	(1.9)			
Other	5	(9.3)			
Time since LT, days (range)	2010	(103–7773)			
Immunosuppression, n (%)	(data missing from 2 patients)				
Tacrolimus	43	(79.6)			
Sirolimus	2	(3.7)			
Ciclosporine	4	(7.4)			
Corticosteroid	22	(40.7)			
Mycophenolate	14	(25.9)			
Unknown	2	(3.7)			
Post-transplant biopsy available, n (%)	31	(57.4)			
Episode of rejection, n (%)	5	(9.4)			
Patients with HCV					
Number treated for HCV, n (%)	5/18	(27.8)			
Successful treatment, n (%)	0/5	(0)			
Viraemic, n (%)	18/18	(100)			
Mean viral load IU/mL (range)	3.59×10^6^	(8.25×10^3^–1.08×10^7^)			
Lab Data (range, SD)					
AST, IU/L	38.6	(16–119, 25)			
INR	1.1	(0.89–2.71, 0.49)			
Albumin, g/L	39.9	(24–46, 4.6)			
Bilirubin, μmol/L	11.4	(3–50, 8.6)			
Platelets, ×10^9^/L	236.9	(73–530, 105)			
Tacrolimus level, ng/mL	5.11	(0.5–14.9, 2.7)			

ALD, alcohol-related liver disease; AIH, autoimmune hepatitis; ALF, acute liver failure; AST, aspartate transaminase; INR, international normalised ratio; NASH, non-alcoholic steatohepatitis; PBC, primary biliary cirrhosis; PSC, primary sclerosing cholangitis.

### Lymphocyte phenotyping

Peripheral blood mononuclear cells (PBMCs) were isolated by density gradient centrifugation and stored in liquid nitrogen. NK cell purification was performed with the Dynabeads Untouched Human NK cells kit (Life Technologies, Paisley, UK). Antibodies used were: CD56-PECy7, CD3-PerCP, CD16-APC-Cy7, CD57-Pacific Blue (all BioLegend, San Diego, USA), CD56-fluorescein isothiocyanate (FITC) (BD Biosciences, Oxford, UK), CD3-Pacific Blue (eBioscience, Hatfield, UK), CD158a,h-PE, CD158b-FITC, CD158b1/b2,j-PE (all Beckman Coulter, Marseille, France), NKp30-APC, NKp46-APC, NKG2D-APC (all Miltenyi Biotec, Gladbach, Germany), NKG2C-PE, NKG2C-PerCP, NKG2C-APC (all R&D Systems Europe, Oxford, UK), rabbit STAT4 (Invitrogen, Paisley, UK) with goat anti rabbit secondary-APC (Abcam, Cambridge, UK).

### Functional assays

#### Degranulation and intracellular cytokine assays

PBMCs were stimulated overnight with 1 ng/mL interleukin (IL) 15 (R&D). These were then incubated with K562 cells at an Effector:Target (E:T) ratio of 10:1 in the presence of CD107a-AF647 antibody (eBioscience), with Golgi Stop (BD Biosciences) and stained with CD3-Pacific Blue and CD56-PECy7 or with anti-interferon (IFN)γ-FITC and antitumour necrosis factor (anti-TNF)α-PE (BioLegend).

#### Cytotoxicity assay

PBMCs were stimulated with 1 ng/mL IL-15, and incubated with K562 cells, prelabelled with CellTracker Orange (Life Technologies), at an E:T ratio of 5:1. K562 cells without effector cells were used as a control. The cells were stained with Live/Dead fixable far-red stain (Life Technologies) and analysed by flow cytometry. Specific killing of the target cells was calculated as follows:
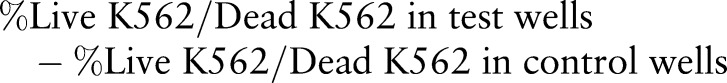


#### Intracellular pSTAT assay

PBMCs were stimulated with 10 ng/mL IL-12 for 60 min. pSTAT4 was measured using the Phosflow kit (BD Biosciences) according to the manufacturer's protocol.

### Immunosuppression assays

PBMCs from healthy donors were incubated in 10% fetal calf serum (FCS) in Roswell Park Memorial Institute (RPMI) 1640 (Biowhittaker, UK) with 1% penicillin/streptomycin, 2 mM glutamine supplemented with 1 ng/mL IL-15, and either tacrolimus (FK506), ciclosporine A or prednisolone (all Sigma Aldrich, Dorset, UK) at the indicated concentrations.

### HLA and KIR genotyping

These were performed as previously described.^25^ ^26^

### Microarray

RNA was extracted from purified NK cells using TRIzol (Invitrogen, Paisley, UK). Hybridisation was performed according to the Agilent 60-mer oligo microarray (Agilent Technologies, Santa Clara, USA). Fluorescence signals of the hybridised Agilent microarrays were detected using Agilent's Microarray Scanner System (Agilent Technologies). The Agilent Feature Extraction Software was used to process the microarray image files. Pathway and functional analyses were performed using Ingenuity Pathway Analysis V.6.0 (Ingenuity Systems, http://www.ingenuity.com). Microarray data are available in the ArrayExpress database under accession number E-MTAB-2132 (http://www.ebi.ac.uk/arrayexpress).

### Quantitative real time reverse transcription PCR

RNA was extracted from purified NK cells and the expression of candidate genes was analysed using a custom array plate with wells preloaded with primers (RT^2^ profiler PCR array, Qiagen, UK). Expression of glyceraldehyde-3-phosphate dehydrogenase (GAPDH) and smooth muscle actin (SMA) was assayed for comparative quantitation. MicroRNA-155 (miR-155) quantitative PCR was performed with the miScript PCR system (Qiagen) according to the manufacturer's instructions. Human RNU6B (RNU6-2) miScript primers were used for normalisation controls. Reaction mixes were prepared in triplicate.

### Statistical analysis

Statistical analysis was performed by two-tailed Student's t test or one-way analysis of variance (ANOVA) as appropriate using GraphPad Prism 6 (GraphPad Software, La Jolla, USA). Analyses of the microarray gene expression data were performed with the software environment R together with packages available as part of the Bioconductor project (http://www.bioconductor.org). Raw data was quality assessed using scatter plot matrices, box plots and principal components analysis. Replicate probes were mean summarised and quantile normalised using the preprocess Core R package. The limma R package^27^ was used to compute empirical Bayes moderated t statistics to identify differentially expressed genes between groups. A corrected p value cut-off of <0.05 was used to determine significant differential expression. The average power for the analysis was 0.81, varying between 0.40 (for lysosome associated membrane protein (LAMP) assays) and 0.99 (for natural cytotoxicity receptor (NCR) expression) for significance at the 5% level.

## Results

### Downregulation of activating receptors on NK cells in non-HCV transplant recipients

**Figure 1:**
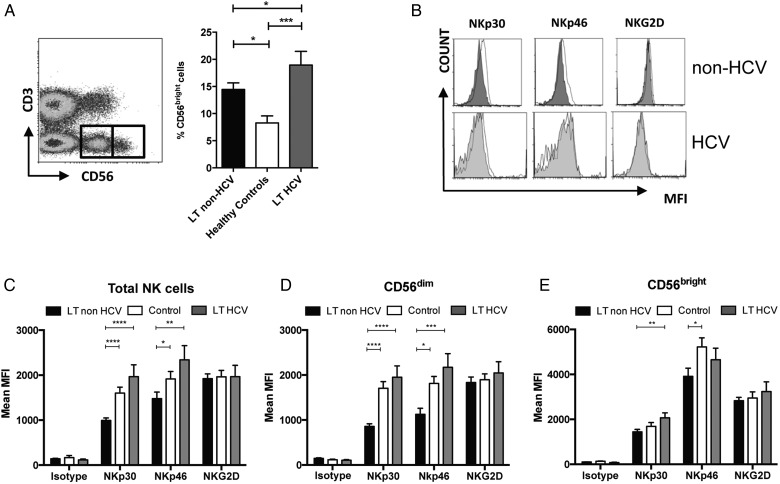
Phenotypic changes in natural killer (NK) cells post liver transplantation (LT). (A) CD3^−^/CD56^+^ NK cells were gated and separated into CD56^dim^ (left-hand square) and CD56^bright^ (right-hand square) subpopulations prior to analysis. The percentage of CD56^bright^ NK cells is shown in the bar chart. (B) Representative histogram plots demonstrating the mean fluorescence intensity (MFI) of the activating receptors in patients with HCV and non-HCV LT (filled histograms) as compared with healthy controls (open histograms). (C–E) Comparison of expression of NKp30, NKp46 and NKG2D on total NK cells (C) and CD56^dim^ (D) and CD56^bright^ (E) NK subsets. For all charts mean and SEM are shown (*p<0.05, **p<0.01, ***p<0.001, ****p<0.0001).

NK cell phenotyping was performed on PBMCs from 54 LT individuals and 31 healthy controls. As chronic HCV infection can affect NK cell phenotype and function we divided the cohort into those transplanted for HCV (n=18) and those for other diseases (n=36) (table 1). NK cells were divided into CD56^bright^ and CD56^dim^ subsets and expression of the activating receptors NKp30, NKp46 and NKG2D analysed (figure 1). The proportion of CD56^bright^ NK cells was increased in the LT cohort as compared with healthy controls (15% vs 8%, p<0.001). This was observed in the HCV positive (19% vs 8%, p<0.001) and non-HCV (13% vs 8%, p=0.011) recipients (figure 1A). The increase in CD56^bright^ NK cells in HCV versus non-HCV LT (19% vs 13%, p=0.012) is consistent with previous studies of NK cells in non-transplant chronic HCV infection.^28^

We found that NKp30 and NKp46, but not NKG2D, were downregulated on NK cells post LT (NKp30 p<0.001, NKp46 p<0.05, figure 1B, C) and that this was confined to individuals not transplanted for HCV infection. Furthermore downregulation was more notable on the CD56^dim^ subset of NK cells, with NKp30 downregulated more than 50% (figure 1D, E). There was no difference in NCR expression on CD158a and CD158b expressing NK subsets, suggesting that the effects seen are not be related to HLA-C allospecificity (see online supplementary figure S1). The disparate findings for NK cells in HCV and non-HCV LTs are consistent with observations of upregulation of NK cell activating receptors in chronic HCV infection in untransplanted individuals and previous work in individuals transplanted for HCV-associated cirrhosis.^17^ ^29–31^

10.1136/gutjnl-2015-309395.supp1 Supplementary figures

### NK cells in LT recipients are hypofunctional

**Figure 2:**
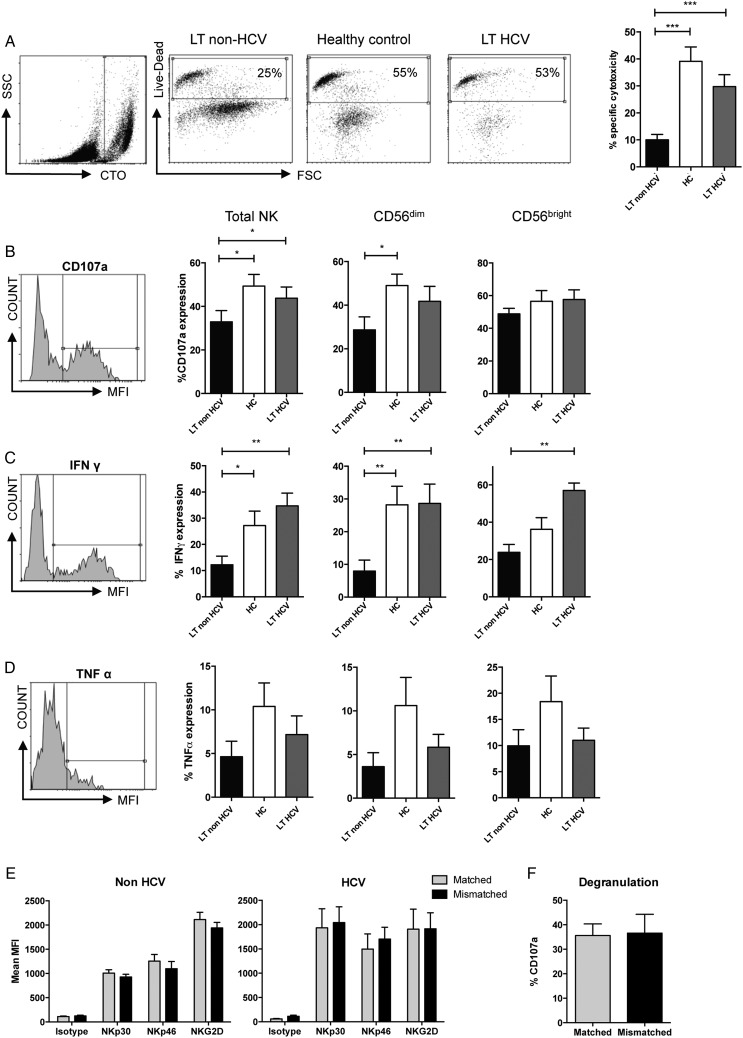
Hypofunctional natural killer (NK) cells in liver transplantation (LT). (A) Flow cytometry cytotoxicity assays using purified NK cells and K562 targets. Representative CellTracker Orange (CTO) staining of the target cells (far left panel), and cytotoxicity assays (middle panels) from each patient group are shown and the results from 14 LT non-HCV, 9 LT HCV and 22 healthy donors are charted (right). (B–D) Flow cytometry plots (left panels) and summary of CD107a degranulation assays (B), interferon (IFN)-gamma secretion (C), and tumour necrosis factor (TNF)-α secretion (D). (E) Effect of HLA-C matching on expression of activating receptors on NK cells. Grey bars (▪) indicate expression on NK cells expressing an inhibitory killer cell immunoglobulin-like receptor (KIR) for the donor HLA-C and black bars (▪) NK cells expressing a KIR without a ligand in the donor liver. (F) Comparison of CD107a degranulation on matched or mismatched NK cells. For all graphs means and SEM are shown (*p<0.05, **p<0.01, ***p<0.001).

To determine if the downregulation of NKp30 and NKp46 had a functional correlate we measured the killing capacity of NK cells from the LT recipients against MHC class I deficient targets. NK cells were stimulated with IL-15 and K562 targets, which express the ligand for NKp30.^30^ These assays demonstrated that K562 killing (p<0.001) and degranulation as determined by CD107a expression (p=0.035), were both impaired in the non-HCV, but not HCV-positive, recipients (figure 2A, B). Similarly we found an impairment of IFNγ production in these individuals compared with controls (p=0.02, figure 2C). Although we observed a trend towards lower levels of TNFα secretion, this did not reach statistical significance (figure 2D). These functional data are consistent with chronic HCV infection overcoming LT-associated NK cell tolerance and the associated poorer outcome of LT for untreated HCV as compared with non-viral causes.

To investigate if the NK cell changes observed in vivo were related to the direct effects of immunosuppressive medication, we determined the effects of tacrolimus, ciclosporine A and prednisolone on the phenotype and function of NK cells, from five healthy donors. We observed minor effects of prednisolone on NKG2D and NKp30 expression, and of ciclosporine on NKG2D expression (see online supplementary figure S2). However there were no changes in cytotoxicity. Furthermore, in the patient cohort, length of immunosuppression (or time since transplant) was not associated with NCR expression, NK function or advanced fibrosis (see online supplementary figure S3).

These data imply that our observations of NK cells in LT in vivo are not a direct effect of immunosuppression on mature NK cells, but are most likely related to changes affecting developing NK cells, as they develop from CD56^bright^ to CD56^dim^.

### HLA-C mismatch does not influence recipient NK cell alloreactivity

We next investigated if mismatching at HLA-C affected NK cell reactivity by comparing expression of activating receptors on NK cells expressing KIR with a ligand present in both the recipient and donor (matched) versus those with KIR for a ligand in the recipient, but not the donor (mismatched) (figure 2E, F). There was no difference in activating receptor expression between matched and mismatched groups (figure 2E). Similarly there was no effect of HLA-C matching on CD107a degranulation (figure 2F). Thus in our cohort HLA-C mismatching does not affect expression of natural cytotoxicity receptors, or NK cell activity, implying that NK cell tolerance is occurring upstream of MHC-dependent education or ‘licensing’.

### NK cell STAT4 gene expression is downregulated in LT

**Figure 3:**
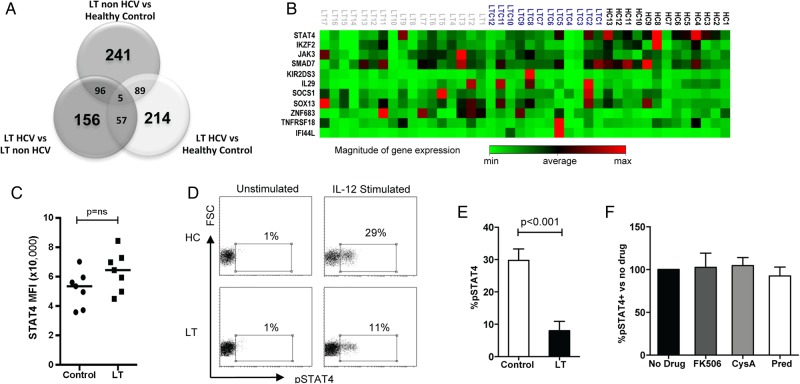
STAT4 gene expression and signalling is altered after liver transplantation (LT). (A) Venn diagram of differentially expressed genes by microarray. Bold type indicate the number of genes differentially expressed between two groups only, and intersections indicate the numbers of genes differentially expressed in common between two or three sets of groups. (B) Clustergram depicting gene expression of candidate genes evaluated by qRT-PCR (HC, healthy control; LTC, HCV LT; LT, non-HCV LT) (C) Summary of basal STAT4 levels in natural killer (NK) cells from LT recipients (n=7) and HCs (n=7) as determined by flow cytometry. The mean fluorescence intensity of STAT4 is plotted. (D) Representative flow cytometry histograms showing interleukin (IL) 12 stimulated pSTAT4 staining in patients with LT and HCs. (E) Summary of pSTAT levels in eight LT and eight control individuals. (F) IL-12 stimulated pSTAT4 levels in peripheral blood mononuclear cells (PBMCs) from three healthy donors when incubated with immunosuppressive drugs for 48 h. Charts show means, SEM and p values <0.05. CysA, ciclosporine; pred, prednisolone.

To investigate potential mechanisms of NK cell tolerance in LT we performed a whole genome microarray experiment, comparing gene expression in NK cells between LT non-HCV, LT HCV and healthy controls (n=4 in each group). Over 800 genes were differentially expressed between the three groups (figure 3A). Eleven significant candidate genes were chosen from the microarray data for validation by quantitative RT-PCR in 13 healthy controls, 17 LT non-HCV, and 12 LT HCV (figure 3B). Significant findings are shown in table 2. The most consistent difference between LT and healthy controls was a downregulation of *STAT4* gene expression. This occurred in both LT groups compared with healthy controls (p=0.0004, −10.73-fold difference, and p=0.01, −3.78-fold difference in LT non-HCV and LT HCV, respectively). Compared with controls, in LT non-HCV there was also upregulation of *ZNF683* (*Hobit*, a repressor of IFNγ expression,^32^ p=0.06, 2.03-fold difference) and downregulation of *KIR2DS3* (p=0.05, −2.14-fold difference). The only candidate gene differentially expressed with near significance between LT HCV and LT non-HCV was *IF144L* (an IFN induced protein, p=0.07, 3.14-fold upregulation in HCV, consistent with the activation of IFN stimulated genes found in chronic HCV infection^33^). When comparing all LTs (HCV and non-HCV) with controls, downregulation of *STAT4* (p=0.0001, −6.97-fold difference) and *IKZF2* (p=0.06, −2.26-fold difference) and upregulation of *ZNF683* (p=0.07, 2.10-fold difference) were found. *IKZF2*, also known as *Helios*, is a transcription factor of the Ikaros family specifically associated with NKp46 expression.^34^ Thus we have observed a trend towards the downregulation of NK cell cytotoxicity and cytokine secretion being determined at a transcriptional level.

**Table 2:** qRT PCR analysis for the candidate genes identified by microarray analysis

	All LT vs HC		LT non-HCV vs HC		LT HCV vs HC		LT HCV vs LT non-HCV	
Symbol	Fold difference	p Value	Fold difference	p Value	Fold difference	p Value	Fold difference	p Value
STAT4	−6.97	**0.0001**	−10.73	**0.0004**	−3.78	**0.01**	2.84	ns
**IKZF2**	−2.26	0.06	−2.88	ns	−1.61	ns	1.79	ns
**ZNF683**	2.10	0.07	2.03	0.06	2.20	ns	1.08	ns
**IF144L**	1.99	ns	1.14	ns	4.38	ns	3.85	0.07
**KIR2DS3**	−2.47	ns	−2.14	0.05	−3.01	ns	0.71	ns

Bold values indicate p<0.05. HC, healthy control; LT, liver transplantation.

However, we found no difference in basal STAT4 levels by flow cytometry, and the Ingenuity Pathway Analysis indicated an effect on JAK/STAT signalling (see online supplementary figure S4). We therefore assessed STAT4 function following stimulation of lymphocytes by IL-12. After IL-12 stimulation, there was significantly lower STAT4 phosphorylation in LT recipients compared with healthy controls (p<0.001) (figure 3C–E). Thus these data demonstrate a functional deficiency in IL-12 signalling related to potentially low levels of an active isoform of STAT4.

In order to exclude this effect as being directly due to immunosuppressive medication we tested the effects of tacrolimus, ciclosporine A and prednisolone on IL-12 induced STAT4 phosphorylation. We observed no change in STAT4 phosphorylation following incubation with immunosuppressants (figure 3F). Thus the tolerant NK cell phenotype is associated with defective STAT4 signalling, and this is not related to the direct effect of immunosuppression on mature NK cells.

**Figure 4:**
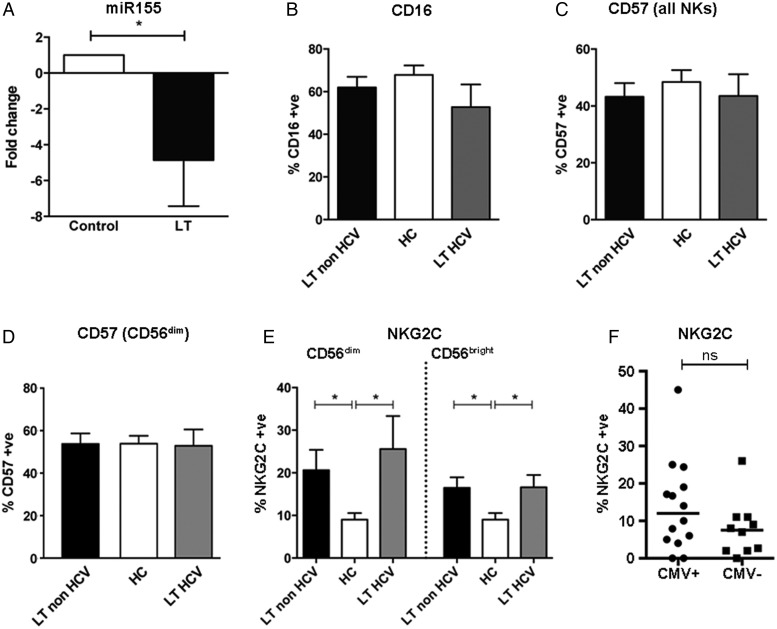
Changes in natural killer (NK) cell maturation markers after liver transplantation (LT). (A) The relative miR-155 level in NK cells from LT recipients (n=7) compared with healthy controls (HCs, n=7) as determined by RT-PCR (means and SEM are shown). (B–F) Comparison of of expression of CD16 on CD56+ NK cells (B), CD57 on CD56+ NK cells (C), CD57 on CD56^Bright^ and CD56^Dim^ NK cells (D) and NKG2C on CD56^Bright^ and CD56^Dim^ NK cells (E) in LT non-HCV (n=20), LT HCV (n=8), and healthy controls (n=14). Charts show mean values and SEM (*p<0.05). (G) Expression of NKG2C on CD56+ NK cells from CMV seropositive (n=14) and CMV seronegative (n=9) LT recipients (ns=non-significant). CMW, cytomegalovirus.

In addition to controlling NK cell activation, STAT4 is also thought to control NK cell differentiation through miR-155.^35^ ^36^ Quantitative RT-PCR performed on purified NK cells from patients post LT and healthy controls demonstrated a 4.9-fold downregulation of miR-155 in LT compared with healthy controls (p=0.04, figure 4A). This is consistent with our observed defect in STAT4 signalling. Thus the effects of *STAT4* downregulation have an ongoing effect on NK cells in post-transplant patients. In mice miR-155 is associated with accelerated NK cell maturation, and deletion of this miRNA has been shown to result in defects in NK cell maintenance and homoeostasis.^36^ We therefore investigated whether equivalent deficits are observed in human LT recipient NK cells by assessing NK cell maturity using the markers CD16, CD57 and NKG2C. These markers have been shown to be associated with terminal differentiation of NK cells and a ‘memory’ phenotype.^37^ ^38^ We found no difference in expression of CD16 or CD57 between LT recipients and healthy controls, and specifically no difference in CD57 expression on CD56^dim^CD16^+^ NK cells between the groups (figure 4B–D). This indicates that the low levels of cytotoxicity observed post LT is not related to accumulation of the hypofunctional CD57+CD16+ NK cell subset. However, a significantly greater proportion of NK cells expressed NKG2C in LT non-HCV only (p=0.019). There was also greater NKG2C expression in CD56^bright^ and CD56^dim^ subsets in both LT groups versus controls (figure 4E). As NKG2C expression has previously been associated with CMV infection,^38^ we compared NKG2C between CMV seropositive and seronegative individuals within our cohort. There was no significant difference between the two groups although there was a trend towards an increase in the CMV seropositive group (14% vs 8% in CMV+ and CMV−, respectively, p=0.38, figure 4F). Thus the increase observed in NKG2C may in part be related to the effects of CMV, but overall we found no specific changes in receptor expression that reflect altered maturation of the CD56^dim^ NK cell subset. Thus overall our data are consistent with changes in NK cells occurring upstream of full functional maturation of NK cells, potentially at the transition between CD56^bright^ and CD56^dim^ NK cells.

## Discussion

We provide an analysis of human NK cells in LT demonstrating changes in phenotype, function and mRNA expression. This tolerant NK cell phenotype has not been previously described and is important in explaining tolerance to liver allografts, but may also have relevance for autoimmune liver disease in which inducing tolerance represents a therapeutic option. Importantly it is significantly different from other transplants, such as stem cell transplantation in which NK cell alloreactivity is observed,^39^ and is consistent with the unique tolerogenic environment of the liver. One possibility accounting for this tolerance is that immature NK cells undergo a maturation step within the liver, as occurs in secondary lymphoid tissue, and that this maturation step is altered by the cytokine microenvironment of the liver. Interestingly, changes in miR155 and NKG2C suggest an effect on NK cell maturation in this context. However, further important insights into this process could be gained by the study of intrahepatic NK cells.

We observed transcriptional level changes in LT, the most significant of which was STAT4 downregulation (table 2 and figure 3). This was not associated with changes in total STAT4 levels as determined by flow cytometry. A lack of correlation between protein and mRNA levels can be related to lower rates of protein as compared with mRNA turnover.^40^ However STATs exist in several different isoforms, including C terminally truncated variants of the mature protein, which act as dominant negatives.^41–44^ As the primers for the qPCR were against the 3' region of STAT4 then potentially the qPCR assay and the flow cytometry assay could be detecting different and non-functional or potentially dominant negative isoforms. Conversely, in the microarray experiment we did not observe changes in molecules that directly control STAT4 function such as SOCS3. Thus further investigation of STAT4 isoforms would be an important area for study of the role of STAT4 in lymphocyte signalling.

In immunogenetic studies STAT4 has been implicated in primary biliary cirrhosis and autoimmune hepatitis.^45^ ^46^ In genome wide association study (GWAS) studies STAT4 and IL-12 single nucleotide polymorphism (SNPs) are associated with primary biliary cirrhosis with extremely high probabilities (both <10^−18^), consistent with this being a key axis in liver inflammation and tolerance.^47^ Similarly STAT4 regulates downregulation of miR155, which is associated with impaired NK cell memory and antiviral responses.^36^ The importance of adaptive or ‘memory’ NK cell responses are being increasingly recognised.^37^ ^38^ Long-lasting memory NK cells have the ability to expand rapidly on repeated exposure to the viral infection. Interestingly this mechanism has been shown to be dependent on IL-12 and STAT4, and deficiencies in either of these results in diminished memory cell expansion.^48^ Therefore, as LT is associated with diminution in IL-12/STAT4 signalling, there may be a similar dysfunction in the generation of longer-lasting memory cells, contributing to the observed hypofunctionality. This could have a beneficial effect in relation to graft tolerance, and a detrimental effect on susceptibility to pathogens, consistent with the accelerated disease progression observed after transplantation for HCV, and for HBV in the prenucleoside era. Furthermore elevated pSTAT4 is associated with a beneficial response to HCV treatment and STAT4 polymorphisms have been associated with accelerated liver fibrosis following transplantation.^49–51^ Thus STAT4 is a key molecule controlling NK cells in liver inflammation and as NK cells can crosstalk with cells of the adaptive immune system, inducing an NK cell tolerant phenotype may be beneficial in suppressing autoimmunity.

Conversely in HCV infection NK cells are less tolerant with relatively normal levels of NKp30 and NKp46 expression, and preservation of cytotoxicity and cytokine secretion (figures 1 and 2). Previous work has demonstrated upregulation of NKp46 in chronic HCV infection^29^ ^52^ ^53^ and one study has correlated higher levels with more severe disease severity.^30^ This suggests that HCV infection is counteracting the effects of downregulation associated with LT. However the downregulation of STAT4 in HCV-LT NK cells indicates that HCV infection circumvents, rather than prevents, NK cell tolerance. Specifically in HCV infection IFNα-stimulated NK cell STAT4 phosphorylation is downregulated with preservation of other pathways.^54^ Using the K562 cell line we were able to test the NKp30 pathway and this appears to have circumvented the observed STAT4 downregulation.

We used K562 cells in combination with IL-15 in assays of NK cell function to assess whether receptor downregulation leads to a functional defect. As K562 expresses ligands for NKp30 and NKG2D, which signal via CD3ζ or FcεRIγ, this assay bypasses the defect in STAT4 observed by us in this study, and others in studies of non-transplant HCV.^49^ ^54^ Thus we observed a downregulation of STAT4 mRNA in our patients with HCV LT as compared with healthy controls, but observed normal levels of IFNγ secretion (figures 2 and 3). In order to further understand the alterations in NK cell functions, and their complex relationship with the discrete effects of transplantation and HCV infection, the study of additional pathways including IFNα and IL-18 would be valuable. However our findings do suggest that several pathways are likely to be affected, as in the patients with non-HCV transplant we have observed hypofunctionality related to receptor-mediated and IL-15 stimulation (figure 2).

The effect on NK cells of immunosuppressive drugs used in transplantation is controversial with investigations to date reporting conflicting results.^20^ ^55^ However in our experiments neither the phenotypic nor the functional findings observed in recipient LT were recapitulated. While there are limitations to these short-term incubation experiments, it is unlikely that the effects we observed in vivo are related to the direct effects of immunosuppressants on mature NK cells. More likely they are due to the complex effects of LT and immunosuppression on NK cell maturation during the CD56^bright^ to CD56^dim^ transition rather than later stage NK cell maturation as we did not observe significant changes in frequencies of CD16 or CD57+ NK cells. These changes likely occur prior to the acquisition of inhibitory receptors for HLA-A, HLA-B and HLA-C. Consistent with this we did not observe any relationship between expression of inhibitory receptors, HLA-C and hypofunctionality. Thus in LT it is unlikely that tolerance of NK cells to the allograft is mediated by KIR:HLA-C interactions, but occurs at a stage prior to the acquisition of KIR. As the phenotypic and functional changes were largely seen in CD56^dim^ NK cells, but were not restricted to NK cells expressing mismatched KIR, our data suggest that recipient NK cells are ‘disarmed’ in a KIR-ligand independent manner. NK cells comprise a higher percentage of the lymphocyte population within the liver compared with the peripheral population and there is increasing evidence that a high proportion of these are phenotypically immature.^56^ ^57^ We therefore propose that immature recipient NK cells become ‘tolerised’ by undergoing an altered maturation process during contact with the allograft, which may be related to immunosuppression affecting the liver cytokine microenvironment or NK cell maturation. Our findings suggest impairment of IL-12 signalling as a potential mechanism for this. While these effects require further investigation, our characterisation of recipient NK cell hypofunctionality provides an insight into the observed tolerability of liver allografts and a model for future studies into NK cell alloreactivity in solid organ transplantation.
