# Supplementary material for: STAT4-associated natural killer cell tolerance following liver transplantation
Source: Gut. 2016 Feb 17;66(2):352–61. doi: 10.1136/gutjnl-2015-309395 (PMC5284485; doi:10.1136/gutjnl-2015-309395)
Supplement: Supplementary figures [file gutjnl-2015-309395supp_figures.pdf]

FIGURE S1

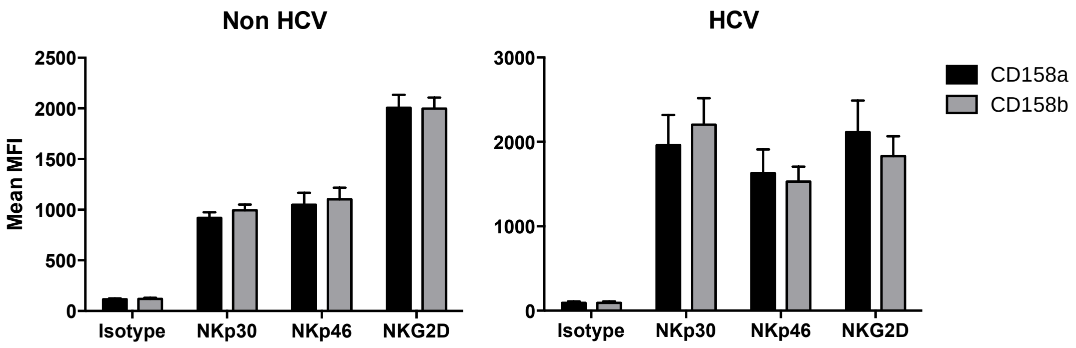

Figure S1: KIR expression on NK cells in LT patients

NK cells from non HCV (n= and HCV LT patients were analysed for CD158a (KIR2DL1/S1) and CD158b (KIR2DL2/3/S2) expression. No significant differences were found.

FIGURE S2

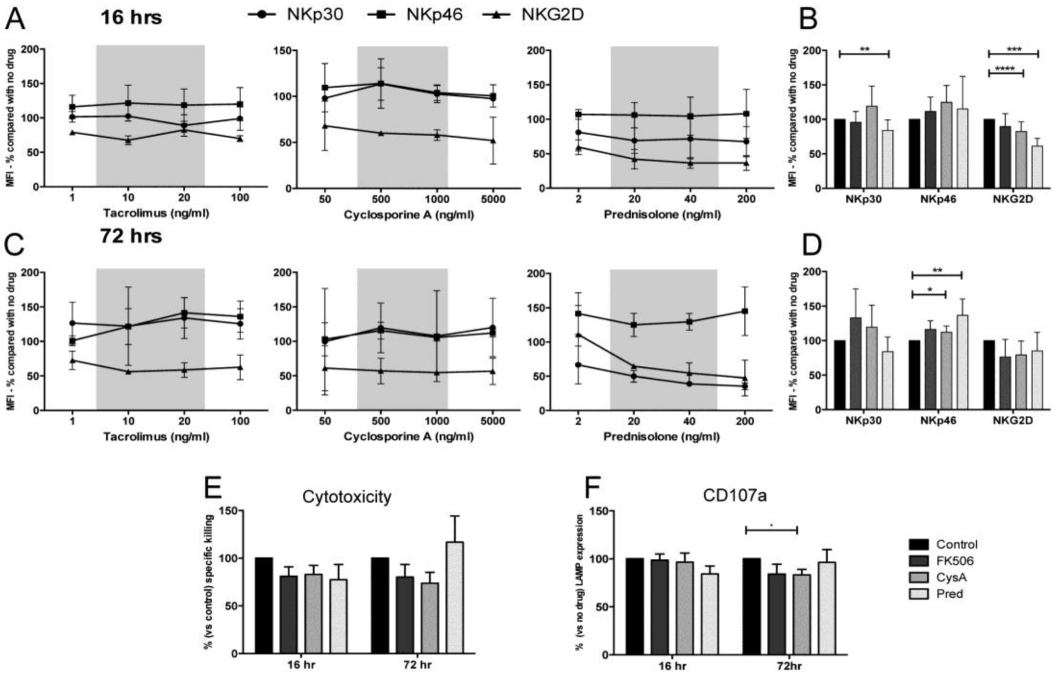

Figure S2: NCR changes are not recapitulated in short term cultures of immunosuppressive agents. Effect of tacrolimus (n=5), cyclosporine (n=4) and prednisolone (n=5) on NK cell activating receptor expression. NK cells from healthy donors were cultured in various concentrations of the different immunosuppressive drugs for 16 hours (**A** and **B**) or 72 hours (**C** and **D**). They were then assessed for activating receptor expression. The effect of drug concentration is shown in panels **A** and **C** (shaded areas indicate usual therapeutic concentrations). A comparison of the different drugs on expression of the individual activating receptors is shown in panels **B** and **D**. **E** and **F** NK cell cytotoxicity (**E**) and CD107a degranulation (**F**) following incubation in the different drugs at mid-therapeutic concentrations. All charts show mean values and SEM (*p<0.05, **p<0.01, ***p<0.001, ****p<0.0001). Key for bar charts: No drug (■) ; Tacrolimus (FK506 ■) ; Cyclosporin A (CysA ■); Prednisolone (pred □).

FIGURE S3

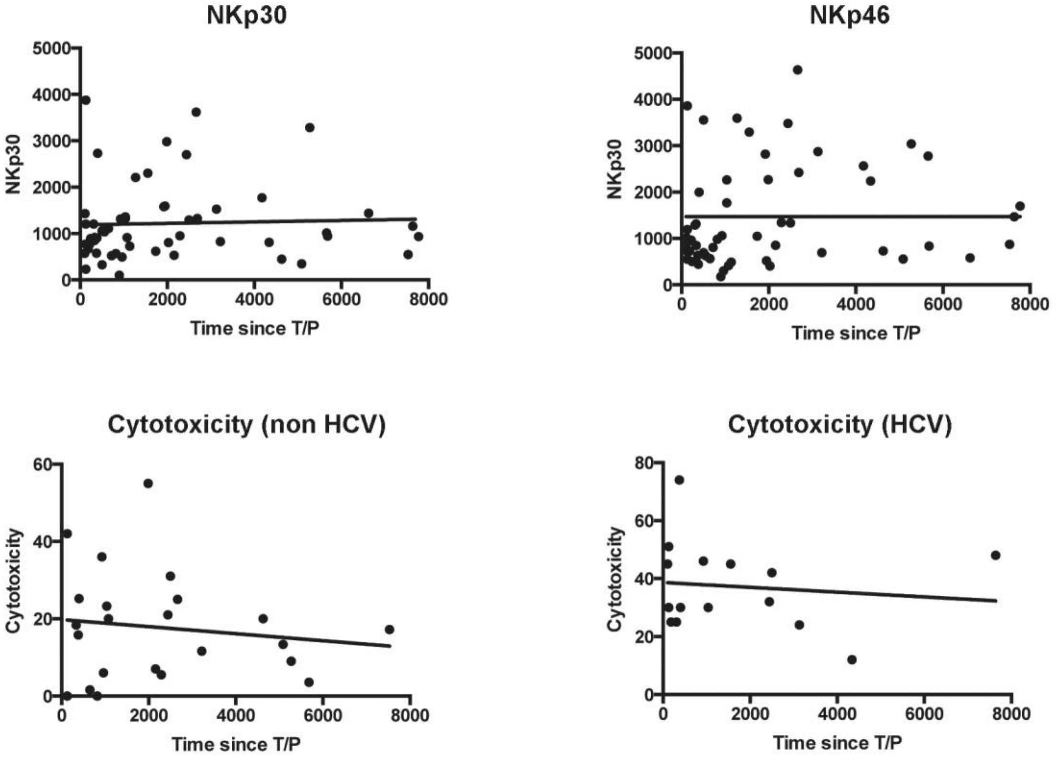

Figure S3: Comparison of time since transplant with NK cell receptor expression and cytotoxicity. No significant correlations were found (p>0.1)

FIGURE S4

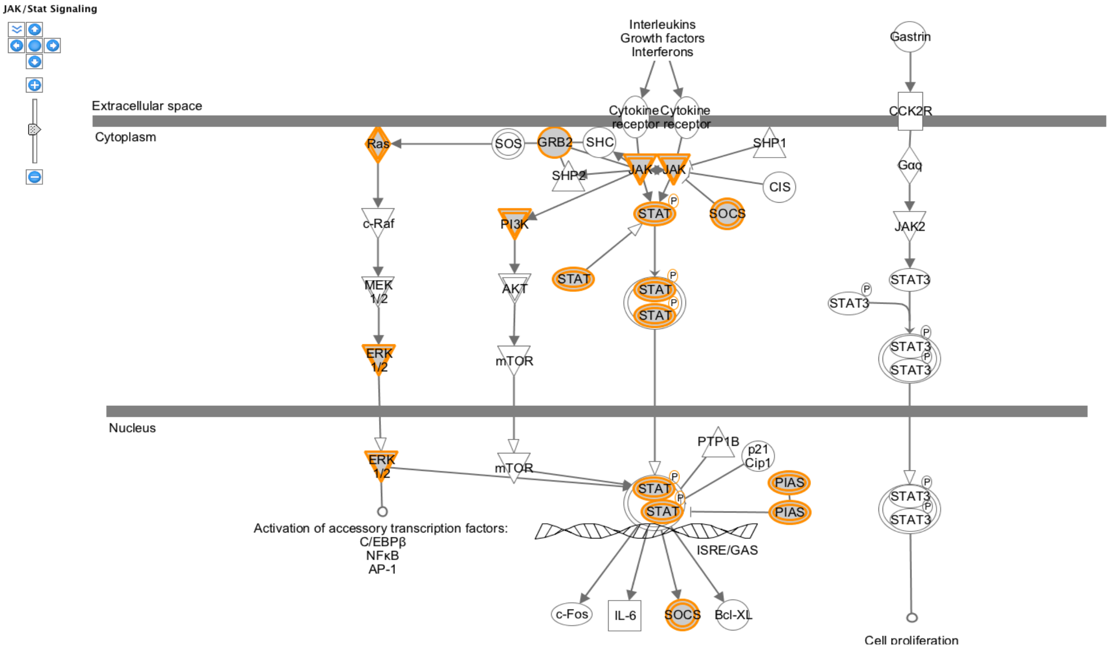

Figure S4: Ingenuity pathway analysis of microarray data with genes in the JAK/STAT pathway affected by liver transplantation highlighted in orange.
